# *In Vivo* Inflammation Does Not Impair ABCA1-Mediated Cholesterol Efflux Capacity of HDL

**DOI:** 10.1155/2012/610741

**Published:** 2012-04-24

**Authors:** Remco Franssen, Alinda W. M. Schimmel, Sander I. van Leuven, Simone C. S. Wolfkamp, Erik S. G. Stroes, Geesje M. Dallinga-Thie

**Affiliations:** ^1^Department of Vascular Medicine, Academic Medical Center Amsterdam, 1105 AZ Amsterdam, The Netherlands; ^2^Department of Experimental Vascular Medicine, Academic Medical Center Amsterdam, 1105 AZ Amsterdam, The Netherlands; ^3^Department of Gastroenterology, Tytgat Institute for Liver and Intestinal Research, Academic Medical Center Amsterdam, 1105 AZ Amsterdam, The Netherlands

## Abstract

HDL provides atheroprotection by facilitating cholesterol efflex from lipid-laden macrophages in the vessel wall. In vitro studies have suggested impaired efflux capacity of HDL following inflammatory changes. We assessed the impact of acute severe sepsis and mild chronic inflammatory disease on the efflux capacity of HDL. We hypothesize that a more severe inflammatory state leads to stronger impaired cholesterol efflux capacity. Using lipid-laden THP1 cells and fibroblasts we were able to show that efflux capacity of HDL from both patients with severe sepsis or with Crohn's disease (active or in remission), either isolated using density gradient ultracentrifugation or using apoB precipitation, was not impaired. Yet plasma levels of HDL cholesterol and apoA-I were markedly lower in patients with sepsis. Based on the current observations we conclude that inflammatory disease does not interfere with the capacity of HDL to mediate cholesterol efflux. Our findings do not lend support to the biological relevance of HDL function changes *in vitro*.

## 1. Introduction

High-density lipoprotein (HDL) cholesterol levels exhibit strong inverse relations with cardiovascular disease risk [1–3]. In the light of this, raising HDL has been a prime target in current drug therapy. HDL exerts a variety of antiatherogenic effects [4]; it improves endothelial function, inhibits thrombosis, and has potent antioxidative properties as well as anti-inflammatory effects [5–7]. Most importantly, HDL is a key player in reverse cholesterol transport (RCT), a process that facilitates transport of cholesterol from peripheral tissues to the liver [8, 9]. In the course of this process, small HDL precursors, such as lipid-poor apoA-I and pre-*β* HDL, acquire free cholesterol and phospholipids through the action of ATP binding cassette transporter A1 (ABCA1) [10]. Maturation occurs through the action of lecithin cholesterol acyltransferase (LCAT), and further lipidation of these particles occurs by interaction with the ATP-binding cassette transporter G1 (ABCG1) resulting in the formation of *α*-HDL particles [11]. Finally, HDL lipoproteins are—as described for mice—directly taken up in the liver by scavenger-receptor-B1 (SR-B1) or HDL lipids are transferred to apo-B-containing lipoproteins via the action of cholesterol ester transfer protein (CETP) and will be subsequently cleared through hepatic low-density lipoprotein (LDL) receptors [5].

More recently, concerns have been raised that, beyond HDL quantity, HDL quality changes may also contribute to impaired cardiovascular disease (CVD) protection [12]. Inflammation is a central feature during all stages of atherosclerotic plaque formation with cytokines and chemokines orchestrating the influx of immune cells in disease vessels [12, 13]. At the same time, cellular activation status of local macrophages determines oxidation of subintimal cholesteryl esters and plaque stability [14]. Systemic inflammatory disease, such as rheumatoid arthritis, has been associated with an increased CVD risk [15]. Patients with active Crohn's disease (CD) also presented with accelerated atherogenesis [16], although a recent meta-analysis was unable to substantiate an increased CV mortality rate in CD [17].

Recent *in vitro* findings suggested that the propensity towards CVD risk in chronic inflammatory disease might in part be related to an impaired protective capacity of HDL particles. It has been shown that myeloperoxidase (MPO), a potent inflammatory mediator predominantly derived from polymorphonuclear cells, can modify apoA-I, resulting in the generation of “dysfunctional” HDL particles with attenuated capacity to mediate ABCA1-dependent cholesterol efflux [18–22]. Colocalization of MPO and apoA-I in the human plaque has fuelled the concept that MPO may also be involved in HDL modification *in vivo* [20, 23, 24].

In the present study we set out to analyse whether systemic inflammatory disease, dependent upon the severity of the disease, affects the capacity of HDL to mediate cholesterol efflux. HDL from patients in a mild chronic (Crohn's disease—CD) and acute severe inflammatory state (sepsis) was tested in *in vitro* cell-based efflux systems. A fibroblast cell line, expressing only ABCA1 was used to test the initial hypothesis that inflammation may specifically affect apoA-I-mediated cholesterol efflux. THP1 cells, a macrophage cell line expressing ABCG1, ABCA1, and SR-B1, were used to reflect total *in vivo* cholesterol efflux. These studies may imply that quantity of HDL may be more important for total cholesterol efflux than the quality of HDL. 

## 2. Methods

### 2.1. Patients

Ten patients with Crohn's disease (CD) were recruited at the outpatient clinic (inflammatory bowel disease) at the AMC. During study visits, disease activity was assessed using the Harvey Bradshaw index (HBI, a research tool taking into account clinical parameters such as, general well-being, abdominal pain, number of liquid stools per day, abdominal mass and complications). Five patients with HBI > 4 were considered to have active CD and five patients with HBI < 4 were in remission. Additionally, we have selected 8 patients with severe sepsis from the ICU unit of the AMC. Sepsis was defined as the presence of a hemodynamic compromised state with temperature above 38°C and CRP levels above 50 mg/L. Additionally 5 healthy controls were recruited from the general population. The institutional review board at the AMC approved the study. All subjects gave written informed consent. 

### 2.2. Blood Collection and Biochemical Measurements

Blood was collected after an overnight fast in EDTA-containing tubes and directly placed on ice. Blood of the patients with sepsis was collected during the admission to the hospital disregarding the fasting status of the patient. Plasma was isolated by centrifugation at 4°C, 3000 g for 15 minutes and stored at −80°C for further analyses. Cholesterol and triglycerides were analysed using a commercially available enzymatic method (Randox, Crumlin, UK) on the Cobas Mira autoanalyzer (Roche, Basel, Switzerland). HDLc and LDLc were analysed by a commercial assay (WAKO, Neuss, Germany). ApoB and apoA-I were analysed using a turbidimetric assay (WAKO, Neuss, Germany). Plasma C reactive protein (CRP) was analysed with a high-sensitivity nephelometric assay (Roche, Basel, Switzerland). Serum amyloid A (SAA) concentration was analysed with a commercial available ELISA (EL10015) from Anogen (Mississauga, Canada). HDL preparations of all patients were included in the efflux studies. 

### 2.3. HDL Isolation

HDL (*d*: 1.063-1.21 g/mL) was isolated by gradient ultracentrifugation in a TLR 100.4 rotor (Beckman Coulter, Fullerton, USA). In short, 0.9 mL human plasma was brought to density *d* = 1.31 g/mL by addition of KBr. UC-tubes were filled with 4 ml 0.9% NaCL. 0.9 ml high density plasma was loaded underneath the NaCL using large injection needles. HDL fraction was harvested after centrifugation for 2 h, 12°C, 100,000 rpm in a Beckman Table top ultracentrifuge. HDL was subsequently dialysed against PBS (0.9% NaCL, 10.9 mmol/L Na_2_HPO_4_·2H_2_O, 1.8 mmol/L NaH_2_ PO_4_·2H_2_O, pH 7.4; Fresenius Kabi), and apoA-I concentration was measured and used directly in the efflux experiments. Then, HDL was obtained by precipitation of the apoB-containing lipoprotein fraction with polyethylene glycol 8000 (Sigma P-2139) as described [25].

### 2.4. Preparation of MPO-Modified HDL

HDL was purchased from Calbiochem (cat. nr. 178452) and used for the MPO-modification reaction exactly as described [19]. In short, HDL (1 mg/mL apoA-I) was added to the reaction mixture containing 50 mM phosphate buffer, pH 7.0, containing 100 *μ*M DTPA, 100 *μ*M NaCl, 140 *μ*M hydroperoxide, 57 nM purified human MPO (BioDesign Int; cat and A63100H). Incubations were performed at 37°C for 1 h. MPO-modified HDL was dialyzed against PBS and used as a positive control in the efflux experiments. 

### 2.5. HDL-Mediated Cholesterol Efflux from Fibroblast and THP1 Cells

Primary human fibroblasts (NHDF-Ad- dermal fibroblasts; Cambrex, CC-2511) expressing human ABCA1 were grown in Hanks Balanced Salt Solution (HBSS) medium without Ca and Mg, containing 10% FBS (GIBCO 14170-088) and 0.1% penicillin/streptomycin at 37°C under 5% CO_2_. To measure cellular cholesterol efflux, fibroblasts were plated and grown until 80% confluency; at this point cells were labelled for 24 h, with 0.5 *μ*Ci/mL ^3^H-cholesterol in labeling medium (D-MEM : F-12  (1 : 1), Glutamax, 0.1% penicillin/streptomycin, 0.2% BSA, and 30 *μ*g/mL cholesterol in the presence or absence of 3 *μ*M LXR agonist T0901317 (Cayman Chemicals Comp.; art 71810) for fibroblasts. After labeling, efflux was induced by the addition of 0.5 mL of efflux medium, consisting of D-MEM : F-12 (1 : 1), Glutamax, 0.1% P/S, 0.2% BSA, and the patients HDL, or HDL from control subjects, isolated by apoB precipitation or HDL isolated by ultracentrifugation at a concentration of 10 *μ*g/mL apoA-I, or HDL isolated from plasma obtained from controls, for a period of 4 h. Efflux medium without apoA-I was used as a negative control, and commercially available apoA-1 at a concentration of 10 *μ*g/mL was used as a positive control. At the end of the incubation period, medium was expired. Cells were extracted with 2-propanol. Both medium and cell extracts were transferred into a vial containing scintillation fluid and counts were assessed (Packard, Tri-Carb 2900TR Liquid scintillation analyzer). Fractional cholesterol efflux was obtained by measuring the release of radio-labeled cholesterol into the medium. The percentage efflux equals the counts in medium divided by total counts in medium and cells combined, subtracted by counts to medium without HDL (control).

THP1 cells (ATCC) were grown in RPMI 1640, glutamax, 25 mM HEPES, 10% FCS, and 0.1% Penn/Strep at 37°C under 5% CO_2_. One day prior to the experiment, cells were differentiated into macrophages by addition of phorbol 12-myristate 13-acetate (PMA: 50 ng/mL; Sigma p8138) to the cells. The efflux procedure was similar to that described above for fibroblasts. All experiments were performed in triplicate on the same day in different wells.

### 2.6. Statistical Analysis

All data are presented as mean (±SD) unless stated otherwise. Statistical analysis was performed in SPSS version 16. All differences in sepsis and controls were analyzed using unpaired Student's *t*-test statistics. The differences in CD-active, CD-remission, and controls were analyzed using two-way ANOVA and LSD post hoc analysis. A *P* value <0.05 was considered as being statistical significant.

## 3. Results

### 3.1. Baseline Characteristics

**Figure 1:**
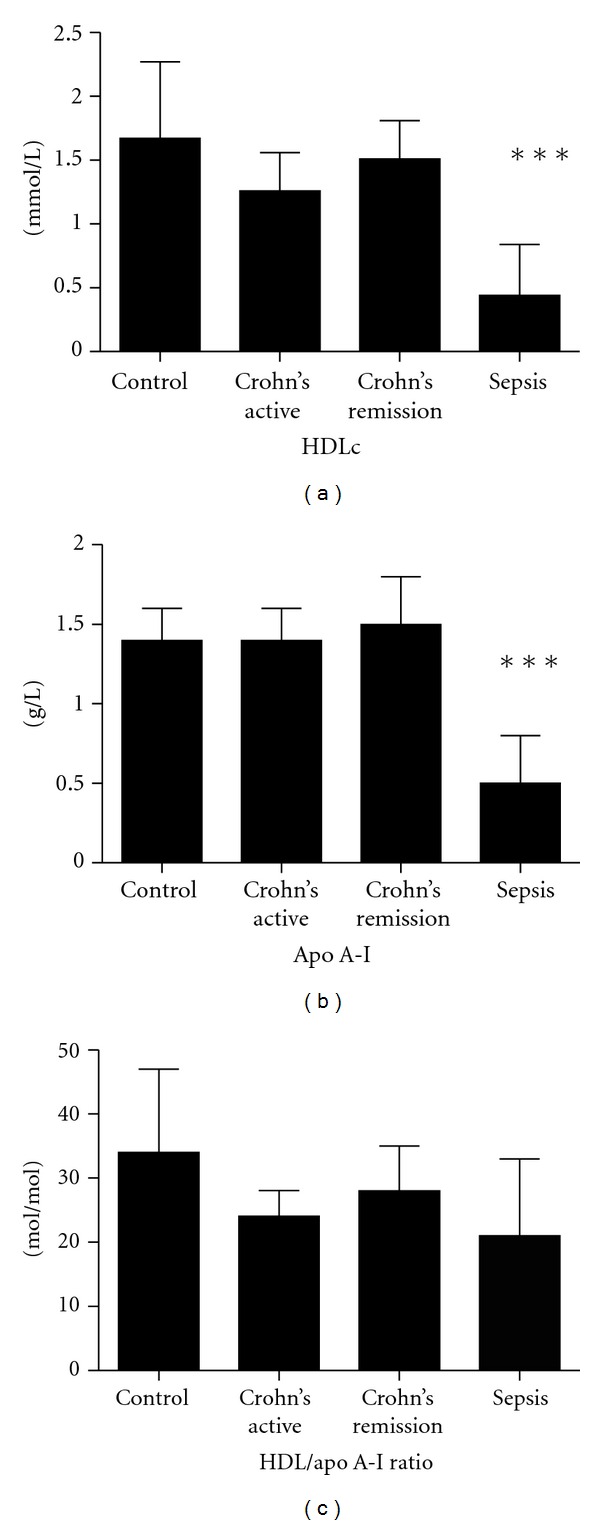
Plasma HDLc and ApoA-I levels in patients with CD, sepsis, and controls. Patients with CD exacerbation had decreased HDLc levels, whereas plasma apoA-I levels were normal. In patients with sepsis, plasma HDLc levels are strongly decreased by 73% and plasma apoA-I levels decreased by 64% as compared to controls. **P < 0.001, ***P < 0.0001.

 The baseline characteristics of patients with Crohn's disease are shown in Table 1. Patients with CD-active, reflecting a mild inflammatory state, had 10-fold higher plasma CRP levels than those with Crohn's disease in remission (*P* < 0.05; Table 1). In line, patients with CD-active had a tendency to lower plasma HDL levels (CD-active: 1.25 ± 1.2 mmol/L versus controls: 1.6 ± 0.6 mmol/L; *P* = 0.11; Figure 1). ApoA-I levels were similar to those in controls. As a consequence, the HDLc/apoA-I ratio in patients with active Crohn's disease was not significantly different (Figure 1).

In patients with sepsis, plasma CRP levels were very high (>200 mg/L). Subsequently, plasma HDLc levels were strongly decreased (0.44 ± 0.4 mmol/L; controls: 1.67 ± 0.6 mmol/L; *P* < 0.001; Figure 1). Plasma apoA-I levels were 64% lower than in controls (0.5 g/L versus 1.4 g/L; *P* < 0.0001); (Figure 1), reflecting a strong decrease in circulating HDL particles. The HDLc/apoA-I ratio was markedly reduced as compared to controls (21 ± 12 mol/mol versus 34 ± 13 mol/mol; *P* < 0.05; Figure 1). Plasma SAA levels in patients with sepsis were significantly higher than in controls (sepsis: >400 mg/L and controls: 2.5 (range: 1.0–7.2) mg/L). Plasma SAA levels in CD-active and CD-remission were measured in a different, but comparable cohort of patients. Plasma SAA levels in CD-active was 23 mg/L (range 3–148) and in CD-remission 5.4 mg/L (range 1.2–69). 

### 3.2. MPO-HDL Displays Impaired Cholesterol Efflux Capacity

**Figure 2:**
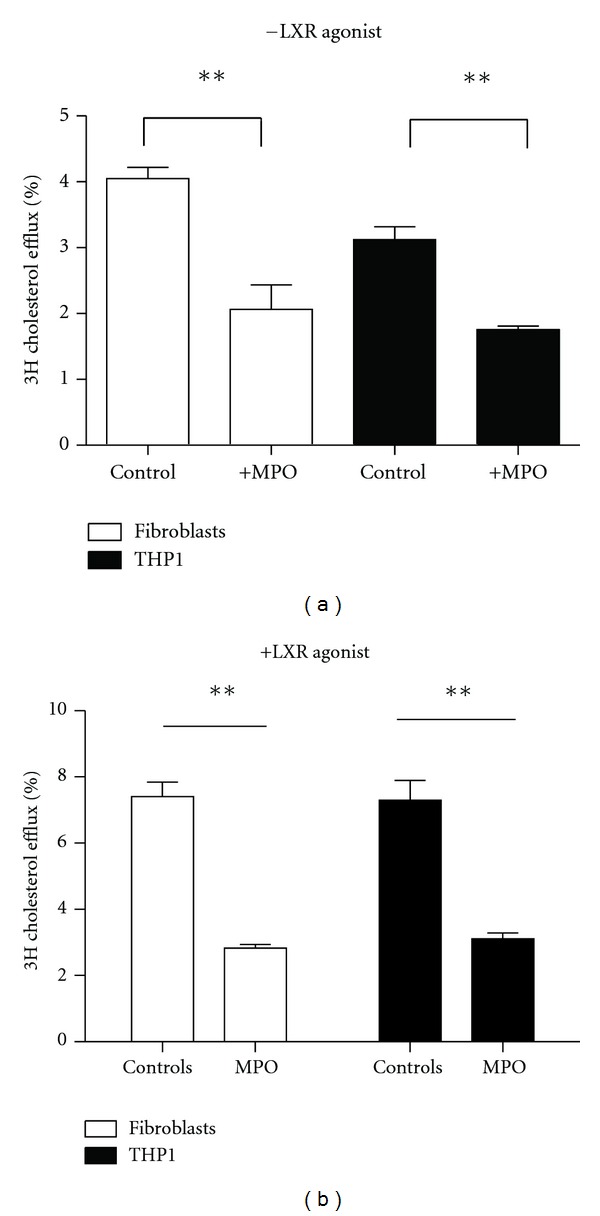
Cholesterol efflux capacity of MPO-HDL in fibroblasts and TP1 cells. HDL and MPO-HDL are used as cholesterol acceptors. Cholesterol efflux potential is measured in fibroblasts (white bars) and THP1 cells (black bars) under cholesterol-loaded conditions as described. The cells are labelled for 24 h, with ^3^H-cholesterol in the absence (a) or presence (b) of LXR agonist. Efflux to HDL (10 *μ*g /mL apoA-I) is measured during a 4 h time span. Efflux medium without HDL is used as a negative control. Fractional cholesterol efflux is determined by measuring the release of radio-labeled cholesterol into the medium. The percentage efflux equals the counts in medium divided by total counts in medium and cells combined, subtracted by efflux to the control. Data are presented as mean ± SD; **P < 0.01. Experiments were performed in triplicate.

 We first tested whether *in vitro* modulation of commercially available human native HDL by MPO impaired the capacity of HDL to mediate cholesterol efflux, measured over a 4 h time span in LXR-stimulated fibroblasts to test ABCA1-mediated efflux. THP1 cells were used to test total efflux capacity mediated by ABG1, ABCA1 and SR-B1 and ABCA1-mediated efflux under LXR agonist stimulation of ABCA1. We have chosen to standardize the amount of HDL added to the incubation system based on apoA-I concentration in the HDL preparations, so that in every efflux experiment comparable amounts of apoA-I are present, reflecting an equal number of HDL particles. In line with expectations, cholesterol efflux capacity of MPO-HDL was significantly decreased by 62% (*P* = 0.008) in fibroblasts and 32% (*P* = 0.01) in THP1 cells (Figure 2) under both conditions, providing evidence that our experimental setup is viable.

### 3.3. Cholesterol Efflux Capacity of HDL from Patients with Crohn's Disease

**Figure 3:**
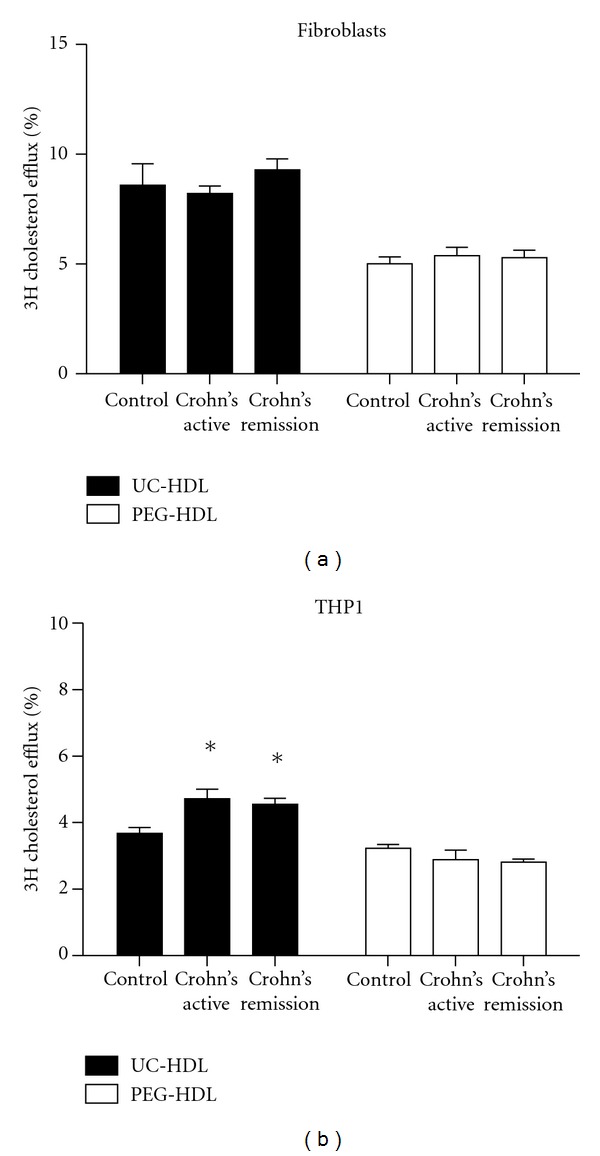
Efflux potential of HDL isolated from patients with Crohn's disease in fibroblasts and THP1 cells. Cholesterol acceptor capacity of UC-HDL (black bars) and PEG HDL (white bars) isolated from controls, and patients with active Crohn's disease and Crohn's disease in remission using cholesterol-laden fibroblasts and cholesterol-laden THP1 cells. Cells are labelled for 24 h with ^3^H-cholesterol. Fibroblasts were incubated in the presence of LXR agonist TO901317. Efflux to HDL (10 *μ*g/mL apoA-I) is measured during a 4 h time span. Efflux medium without HDL is used as a negative control. Fractional cholesterol efflux is determined by measuring the release of radio-labeled cholesterol into the medium. The percentage efflux equals the counts in medium divided by total counts in medium and cells combined and was corrected for counts to medium without acceptor HDL. Experiments were performed in triplicate. Data are presented as mean ± SD; *P < 0.05.

 HDL was isolated by ultracentrifugation as well as by precipitation to avoid bias through the presence of serum proteins in the PEG-precipitated HDL preparation, which may interfere with the cholesterol efflux measurement. Again equal amounts of apoA-I (10 *μ*g/mL) were added to each well in the efflux experiment. Surprisingly, HDL isolated by ultracentrifugation (UC-HDL) showed similar ABCA1-mediated cholesterol efflux in fibroblasts as compared to controls; (Figure 3(a)). UC-HDL showed increased total cholesterol efflux potential in THP1 cells (CD-active: +28%, *P* < 0.02 versus controls; CD-remission: +24%; *P* < 0.01 versus controls (Figure 3(b)). PEG-HDL (10 *μ*g/mL apoA-I), as a control, isolated from CD-active and CD-remission patient's plasma induced a similar ABCA1-mediated cholesterol efflux in fibroblasts as well as total cholesterol efflux potential in THP1 cells as compared to control PEG-HDL (Figure 3).

### 3.4. Cholesterol Efflux Capacity of HDL from Patients with Sepsis

**Figure 4:**
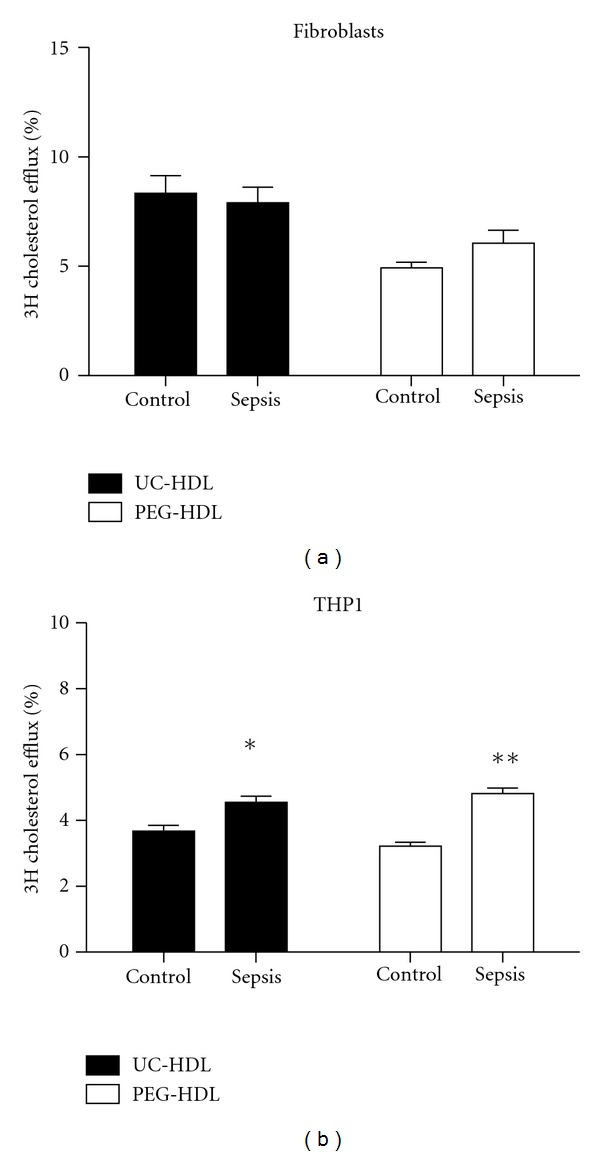
Efflux potential of UC-HDL and PEG-HDL isolated from patients with sepsis in fibroblasts and THP1 cells. Cholesterol acceptor capacity of UC-HDL (black bars) and PEG-HDL (white bars) from patients with sepsis using cholesterol-laden fibroblasts (and cholesterol-laden THP1 cells). Fibroblasts were incubated in the presence of LXR agonist TO901317. Cells are labelled for 24 h with ^3^H-cholesterol. Efflux to HDL (10 *μ*g/mL apoA-I) is measured during a 4 h time span. Efflux medium without HDL is used as a negative control. Fractional cholesterol efflux is determined by measuring the release of radio-labeled cholesterol into the medium. The percentage efflux equals the counts in medium divided by total counts in medium and cells combined and was corrected for counts to the medium without acceptor HDL. Experiments were performed in triplicate. Data are presented as mean ± SD; *P < 0.05, **P < 0.01.

 UC-HDL from patients with sepsis, reflecting a severe inflammatory state, showed a similar ABCA1-mediated cholesterol efflux potential from cholesterol-laden fibroblast cells in the presence of a LXR agonist (Figure 4). Cholesterol efflux potential from THP1 cells to UC-HDL isolated from plasma of patients with sepsis was increased as compared to controls (+14%, *P* < 0.05; (Figure 4). Cholesterol efflux potential of PEG-HDL in cholesterol-laden fibroblast cells was similar to controls (Figure 4), whereas cholesterol efflux capacity of PEG-HDL in THP1 cells was significantly increased (+58%; *P* < 0.005; Figure 4). 

## 4. Discussion

We demonstrate that the capacity of HDL (normalized for apoA-1 concentration) of patients with severe acute or mild chronic inflammatory state, to mediate cholesterol efflux from cultured cholesterol-loaded fibroblasts treated with a LXR agonist to stimulate ABCA1 expression and THP1 cells, was not decreased as compared to normolipidemic controls. Even in patients with sepsis, suffering from a severe inflammatory disease state, HDL efflux capacity is, if anything, increased compared to healthy controls. These data underline that *in vitro* findings regarding HDL “dysfunction” cannot be extrapolated to an *in vivo* situation.

In the present study we have used two different cell systems to determine HDL-mediated cholesterol efflux potential. Fibroblasts are known to express the ABCA-1 receptor abundantly which can be further increased by treating the cells with a LXR agonist, whereas THP1 cells (under lipid-laden conditions) express ABCA1, SR-B1 and ABCG-1 and thus model lipid-laden macrophages in human atherosclerotic plaques [26]. In line with previous reports, we observed that HDL when exposed to MPO in a test tube displays impaired capacity to mediate cholesterol efflux from both fibroblasts as well as THP1 cells, [20, 24] showing that ABCA1-mediated cholesterol efflux is highly impaired and thus provides validation of our efflux system. Two different disease models, one representing an acute, severe inflammatory state (sepsis) and the other representing a mild inflammatory state (Crohn's disease), have been used in the present study. Both disease states were characterized by increased plasma CRP and SAA levels, as well as decreased plasma HDLc levels, albeit not significant in CD-active, which is in line with HDL acting as an inverse acute phase reactant [5]. Our original hypothesis was based on the observation that *in vitro* inflammatory challenges modulate HDL response to induce normal cholesterol efflux from cell culture systems. We expected to see a gradual decrease in HDL efflux potential with increasing severity of inflammatory state. However, despite the inflammatory conditions of these patients we observed a modest increase in the efflux capacity of HDL, but we feel that more robust analysis are required to further investigate this.

Thus, our data clearly indicate that impaired efflux capacity of HDL in inflammatory disease states does not play a significant role *in vivo*. Obviously, these findings do not exclude a decreased total efflux capacity, since HDLc and apoA-I concentrations are profoundly lower in patients with sepsis albeit not in patients with a mild inflammatory state (active Crohn's disease) [15, 27]. The efflux data were obtained through controlling for apoA-I levels in the different HDL preparations. Plasma apoA-I levels in patients with sepsis were 45% reduced. This means in theory that, although HDL was more efficient in accepting cholesterol from cells, this cannot be compensated for by the strong decrease in number of circulating HDL particles and thus an overall decreased total efflux capacity.

Interestingly, we observed a slight increase in HDL efflux capacity in an inflammatory disease state. Van Der Westhuyzen et al. have previously shown that, besides apoA-I, SAA is also capable of promoting cholesterol efflux [28]. Since we have used similar apoA-I concentrations in all our assays and more importantly since HDL particles in the inflammatory state contain an increased number of SAA molecules, this may have been the reason for the increase in efflux capacity. However, plasma SAA levels in patients with sepsis are more than 200-fold increased as compared to controls whereas plasma SAA levels in active Crohn's disease were only increased 10-fold compared to controls. Therefore, we conclude that plasma SAA levels do not contribute to the increased cholesterol efflux observed in both CD-active and sepsis.

Previous studies have suggested that smaller lipid depleted HDL particles serve as better cholesterol acceptors via the ABCA1-mediated efflux pathway [29]. Indeed, the estimated HDL-c/apoA-I ratio between controls and patients in a severe inflammatory state was significantly reduced suggesting the presence of smaller-sized HDL particles. In line, the average number of particles was reduced in our study as reflected by the decrease in apoA-I levels. These data lend further support to the concept that HDL quantity is the main determinant of cholesterol efflux capacity under these conditions. In contrast to our findings, Mc Gillicuddy et al. reported that, following an acute endotoxin challenge, several steps in the RCT pathway were disrupted [30]. Thus, *in vitro* exposure of macrophages to LPS significantly reduced ABCA1 expression thereby attenuating cholesterol efflux capacity towards apoA-I. Cholesterol efflux acceptor capacity of HDL obtained from subjects subjected to the LPS challenge was decreased with a concomitant decrease in plasma apoA-I level. There are several reasons that may underlie the discrepancy with the current findings. First, a single endotoxin challenge is not comparable to a more sustained inflammation, since a wide variety of adaptation mechanisms evolve over time [5, 31, 32]. Most importantly, these authors failed to normalize for the decrease in apoA-I levels after the endotoxin challenge in the cholesterol efflux assay, which invariably contributes to differences in cholesterol efflux. In fact, we demonstrated in septic patients that correction for changes in apoA-I result in a normal or even increased efflux acceptor capacity of HDL independent of the HDL preparation (PEG isolated or ultracentrifugally isolated).

### 4.1. Study Limitations

Our study has several limitations. First, the patients in the present study were older compared to the controls. Berrougui et al. have reported that particularly ABCA1-mediated cholesterol efflux decreased with advancing age [33]. This implies that in patients we may have observed lower values compared to the younger controls. However, since we find, if anything, higher efflux values, the age difference cannot have contributed to the present observation. Second, we included only a limited number of subjects in our study. However, since we observe an increased efflux in patients with an extreme inflammatory disease, it is unlikely that inclusion of a larger number of subjects would alter these findings.

### 4.2. Conclusion

 Whereas exposure of HDL to MPO *ex vivo* leads to an impaired cholesterol efflux, native HDL preparations isolated from patients in mild and severe inflammatory disease states retain its ability to stimulate efflux. These findings do not lend support to the biological relevance of HDL function changes observed *in vitro*.

## Figures and Tables

**Table 1:** Baseline characteristics of the subjects.

	Control	Crohn's disease active	Crohn's disease remission	Sepsis
Age, years	28 (3)	43 (10)	48 (15)	65 (12)*
Male/female	3/2	5/0	4/1	5/3
TC, mmol/L	4.7 (0.6)	4.0 (0.6)	5.12 (1.6)	1.9 (1.0)**
LDLc, mmol/L	2.57 (0.6)	2.14 (0.4)	3.06 (1.4)	1.27 (0.6)**
TG, mmol/L	1.03 (0.5)	1.25 (1.2)	1.22 (0.7)	1.70 (0.7)
CRP, mg/L	0.5 (0.2)	23.5 (27.8)	2.2 (1.8)	282 (109)**

Data are presented as mean (±SD). TC: total cholesterol; LDLc: low density lipoprotein cholesterol; HDLc: high-density lipoprotein cholesterol; TG: triglycerides; apo: apolipoprotein. *P < 0.05; **P < 0.01 versus controls.
